# Geometric and Compressive Characteristics of the Additive-Manufactured Rhombicuboctahedron Structure and Its Application

**DOI:** 10.3390/ma19030619

**Published:** 2026-02-05

**Authors:** Jaerin Kim, Donghyeon Kim, Jeongin Lee, Seong Je Park

**Affiliations:** School of Mechanical Engineering, Gyeongsang National University, 501 Jinju-daero, Jinju-si 52828, Gyeongsangnam-do, Republic of Korea

**Keywords:** additive manufacturing, material extrusion, rhombicuboctahedron, application

## Abstract

In this study, the geometric and compressive characteristics of a rhombicuboctahedron architecture fabricated by material extrusion were investigated. The compressive results showed that increasing the number of unit cells led to the specific compressive strength remaining nearly constant. In contrast, as the strut thickness increased, the structures exhibited higher compressive strength, specific compressive strength, and elastic modulus. In particular, the thickest configuration exhibited no premature fracture or abrupt stress drop, instead demonstrating a progressive densification behavior with continuously increasing stress. Furthermore, a pallet prototype was fabricated to demonstrate practical feasibility. The non-cubic, recessed geometry of the rhombicuboctahedron units enabled geometric interlocking between stacked pallets, increasing surface-induced friction and contributing to enhanced stacking stability and anti-slip performance. These results demonstrate the potential of rhombicuboctahedron architectures as lightweight, scalable, and mechanically reliable structural elements for compression-dominated applications enabled by additive manufacturing.

## 1. Introduction

Geometric structures composed of repeated prismatic units have long attracted interest as mechanically meaningful architectures from a structural mechanics perspective [1,2,3]. A representative example is the Rubik’s Snake, which consists of identical rectangular prisms connected sequentially [4,5]. Although the Rubik’s Snake is commonly recognized as a three-dimensional puzzle, when fully folded into a stable configuration, it forms a regular polyhedral geometry known as the rhombicuboctahedron [6]. The rhombicuboctahedron is one of the Archimedean solids, characterized by uniform vertex configurations and a combination of square and triangular faces [7,8]. Specifically, it consists of eight equilateral triangular faces and eighteen square faces, with identical face arrangements at every vertex [9,10]. This high degree of geometric symmetry suggests that load transfer paths may not be concentrated along a single direction but can be spatially distributed throughout the structure. The geometry comprises prismatic elements arranged along both orthogonal and oblique directions, forming multiple intersecting members. Thus, compressive loads may be redistributed over the entire structure rather than being localized, while the closed-surface topology and repeated junctions may reduce the tendency for localized bending-induced instability and premature failure [11,12,13].

The geometrically complex polyhedral structures, such as the rhombicuboctahedron, may offer high load-bearing efficiency despite relatively low mass, owing to their multiple load-transfer pathways and space-filling characteristics in various industrial applications, including on aerospace structures, mobility systems, energy-absorbing components, and lightweight architectures [14,15]. Other well-established lattice structures, such as cubic, octet-truss, Kelvin, BCC, FCC, diamond, and TPMS-based structures, have been extensively investigated, with well-established correlations between relative density, strut geometry, compressive performance, and industrial applications via additive manufacturing (AM) [16,17,18,19,20,21,22,23,24]. Thus, elucidating the mass-normalized compressive strength and stiffness of the rhombicuboctahedron represents a scientifically and technologically relevant research question using AM.

In the past, the fabrication of complex three-dimensional geometries with interconnected elements has been constrained by manufacturing limitations [25]. Conventional subtractive, forming, or assembly-based processes make it extremely difficult to realize such structures as monolithic components with sufficient geometric fidelity [26,27]. However, the rapid advancement of AM technologies has fundamentally alleviated these constraints [28]. In particular, AM enables the direct fabrication of complex geometries with intricate internal connections without the need for tooling or assembly [29]. This technological shift enables the systematic evaluation of geometry-driven mechanical properties of rhombicuboctahedron structures. By maintaining identical material conditions while varying geometric parameters, the influence of structural arrangement on compressive behavior can be isolated from material effects. Furthermore, quantitative assessment of load-bearing capability normalized by mass allows the lightweight efficiency of the structure to be critically examined.

In this study, rhombicuboctahedron structures are fabricated using material extrusion (MEX), one of the AM technologies, and their compressive behavior is systematically investigated. Specifically, (i) the effect of the number of unit cells on compressive strength is analyzed, (ii) compressive performance is evaluated through fine geometric design tuning of the rhombicuboctahedron architecture, and (iii) the optimized structure is applied to a pallet-type configuration to demonstrate its structural feasibility and application ability. Through these investigations, this work aims to demonstrate the potential of rhombicuboctahedron architectures as lightweight, high-efficiency structural elements and to provide fundamental insights into geometry-based structural design enabled by additive manufacturing.

## 2. Methodology

A MEX machine (A1, Bambu Lab, Shenzhen, China) was used for specimen fabrication. Polylactic acid (PLA) filament with a diameter of 1.75 mm (HS PLA, Hanil Industry Co., Ltd., Namyangju-si, Republic of Korea), in white color, was employed as the MEX material. In this study, the compressive behavior of rhombicuboctahedron-based lattice structures with one unit cell dimension of 13 mm × 13 mm × 13 mm was investigated. To quantitatively evaluate the structural strength, two geometric parameters were systematically varied: the number of unit cells and the strut thickness (Thus, rhombicuboctahedron types). The number of unit cells was set to 1 × 1 × 1, 2 × 2 × 2, 3 × 3 × 3, 4 × 4 × 4, and 5 × 5 × 5, respectively. The strut thickness varied from 1.6 mm, corresponding to the minimum MEX-processable diameter achievable, to 6.5 mm, at which the internal volume of the rhombicuboctahedron unit cell becomes fully filled. In addition, intermediate strut thicknesses of 2.2 mm and 4.3 mm were selected to investigate the effect of gradual changes in structural density. For clarity, the number of unit cells was denoted by a single integer, where 1 × 1 × 1, 2 × 2 × 2, 3 × 3 × 3, 4 × 4 × 4, and 5 × 5 × 5 configurations were labeled as 1, 2, 3, 4, and 5, respectively. Similarly, the strut thicknesses of 1.6 mm, 2.2 mm, 4.3 mm, and 6.5 mm were designated as T1.6, T2.2, T4.3, and T6.5, respectively, to facilitate comparison in the figures and discussions. In the case of T6.5, the unit cell is fully filled. However, as is typical in the MEX process, microscale inter-bead and inter-layer voids may still remain in all specimens. The MEX parameters were set as follows: a layer thickness of 0.2 mm, nozzle diameter of 0.4 mm, nozzle temperature of 220 °C, bed temperature of 55 °C, a nozzle speed of 250 mm/s, raster angle of 45/−45°, and infill density of 100% without any support structure. All rhombicuboctahedron structures were designed using Tinkercad (Autodesk Inc., San Francisco, CA, USA).

To evaluate compressive behavior, a universal testing machine (Instron 34TM-30, Norwood, MA, USA) was used. All compression tests were conducted between the same pair of flat steel platens using an identical testing protocol for all specimen sizes. Specimens were centered on the platen to minimize eccentric loading. Specimens were aligned using the platen center marks (crosshair) and visually confirmed from two orthogonal directions. Compression tests were conducted under dry contact between the steel platens and the specimens, without any lubrication. This ensured consistent frictional boundary conditions across all specimen sizes. All compression tests were conducted at room temperature with a crosshead speed of 5 mm/min and a preload of 5 N. Compression was terminated when the load decreased by more than 30% from the peak load. Specifically, the definitions of stress, displacement, strain, specific compressive strength, and elastic modulus used in this study are clarified as follows. The compressive stress was calculated by dividing the applied load by the overall external dimensions of each specimen. For example, the 1 × 1 × 1 specimen had a reference area of 13 × 13 mm^2^, while the 5 × 5 × 5 specimen had a reference area of 65 × 65 mm^2^. In cases where densification during compression leads to a continuous increase in stress, defining compressive strength based on peak or plateau stress becomes ambiguous. Therefore, in such cases, the compressive strength was determined from the peak load observed before the plateau region. The displacement used in the stress–strain curves corresponds to the crosshead displacement during compression. The strain was defined as the crosshead displacement divided by the initial specimen height, and the value was expressed as a percentage by multiplying by 100, without the use of an extensometer. The specific compressive strength was then defined as the compressive stress divided by this calculated density. The density of each lattice specimen was calculated using the measured mass of the specimen and the overall external dimensions of each specimen. For instance, the 1 × 1 × 1 specimen had a reference volume of 13 × 13 × 13 mm^3^, while the 5 × 5 × 5 specimen had a reference volume of 65 × 65 × 65 mm^3^. The elastic modulus was determined by performing a linear regression on the initial portion of the stress–strain curve. The fitting range was selected within the region where the stress–strain response exhibited a linear trend with a coefficient of determination greater than 0.99, while excluding the initial seating region. All experiments were performed using three specimens for each condition, and the results are presented as mean values with standard deviations (n = 3).

## 3. Results and Discussion

### 3.1. Compressive Behavior According to the Number of Unit Cells

**Figure 1:**
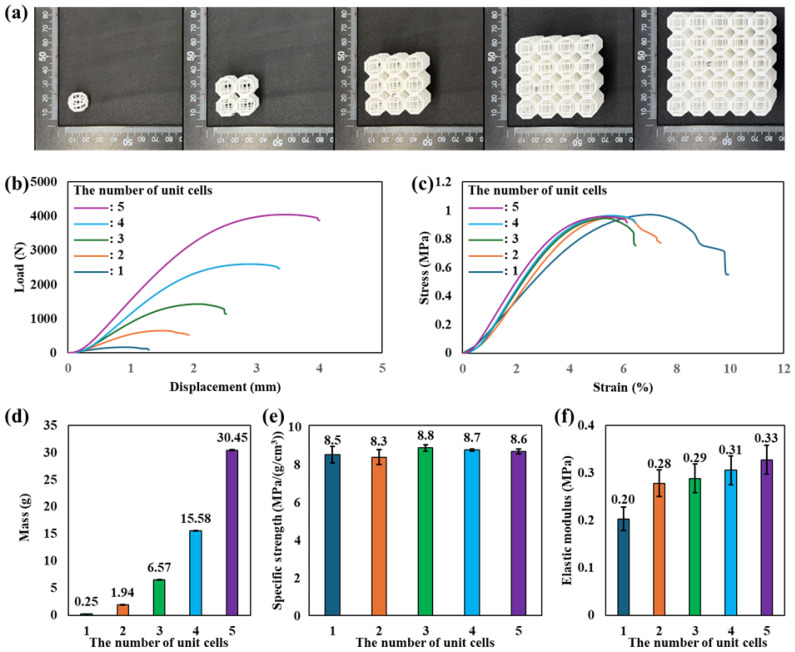
(**a**) Images of MEXed specimens, (**b**) load–displacement curve, (**c**) stress–strain curve, and (**d**) comparison of mass, (**e**) specific strength, and (**f**) elastic modulus for rhombicuboctahedron structures according to the number of unit cells.

Figure 1a shows the MEXed rhombicuboctahedron structures with different numbers of unit cells, ranging from 1 × 1 × 1 to 5 × 5 × 5. All specimens were successfully fabricated using the MEX process without noticeable geometric defects. However, it is well known that MEXed parts inherently contain micro-geometrical imperfections. CT-based studies on PLA-MEX have shown that more than 95% of the geometry remains within ±150 μm deviation from the CAD model (within ISO IT11 tolerance), while most voids are below 230 μm [30]. Such micro-geometrical deviations are closely related to the strand cross-sectional shape formed at the nozzle exit, and recent studies have demonstrated that active control of nozzle size and shape can be used to tailor strand geometry and deposition behavior in MEX, offering a potential pathway to mitigate these inherent errors [31]. The corresponding load–displacement curve is presented in Figure 1b. As the number of unit cells increased, the overall load-bearing capacity of the structure increased monotonically. Specimens with a higher number of unit cells exhibited higher maximum compressive loads and more stable post-yield behavior compared to the single-unit configuration. To eliminate the influence of specimen size and mass, the compressive responses were further analyzed in terms of stress–strain behavior, as shown in Figure 1c. The initial linear elastic regions of all specimens were clearly observed, followed by a gradual deviation from linearity associated with progressive deformation of the strut network. Notably, the stress–strain responses of all configurations exhibited similar ultimate compressive strength levels, indicating that the intrinsic deformation and load-bearing behavior of the rhombicuboctahedron unit cell remains consistent regardless of the number of unit cells. Meanwhile, the strain decreases as the number of unit cells increases, from approximately 10% for 1 × 1 × 1 to about 6% for 5 × 5 × 5. If failure occurred at identical locations for all specimens, such a reduction in strain capacity would not be expected. Specimens with a small number of unit cells are strongly affected by free-surface effects, where reduced constraint and stress-free cell walls cause localized, progressive collapse that permits larger global deformation. Conversely, specimens with many unit cells exhibit uniform load transmission, which leads to more simultaneous collapse and therefore shorter global strain to failure [32]. Figure 1d compares the mass of the specimens as a function of the number of unit cells. As expected, the mass increased approximately proportionally with the number of unit cells, reflecting the systematic addition of material volume. However, when the compressive strength was normalized by density, a different trend emerged. As shown in Figure 1e, the specific compressive strength remained nearly constant across all configurations with different numbers of unit cells. The coefficient of variation (CV%) of the specific compressive strength for each configuration was evaluated to examine the repeatability of the measurements. The CV% was 5.14% for the 1 × 1 × 1 specimen, 4.73% for the 2 × 2 × 2 specimen, 1.95% for the 3 × 3 × 3 specimen, 0.77% for the 4 × 4 × 4 specimen, and 1.45% for the 5 × 5 × 5 specimen. To further verify that the specific compressive strength was statistically independent of the number of unit cells, a one-way ANOVA was conducted using the measured values from each configuration (n = 3 per group). The analysis revealed no statistically significant difference in specific strength across specimen sizes (F = 1.43, *p* = 0.294). This result confirms that the consistent specific strength is not due to experimental scatter but reflects an intrinsic size-independent mechanical efficiency of the rhombicuboctahedron architecture. This result suggests that the rhombicuboctahedron architecture preserves its mass-normalized load-bearing efficiency as the structure scales up, with no statistically significant size effect observed within the tested range [33,34,35]. In other words, the intrinsic mechanical efficiency of the unit cell itself remains unchanged. The consistent specific compressive strength indicates that the rhombicuboctahedron architecture maintains stable load-bearing behavior without introducing structural inefficiencies as the number of unit cells increases. The elastic modulus is summarized in Figure 1f. The elastic modulus increased by approximately 1.65 times as the number of unit cells increased from 1 to 5, indicating enhanced resistance to elastic deformation in larger assemblies. This behavior is attributed to the increased structural continuity and the interaction between neighboring unit cells, which collectively contribute to a higher effective stiffness under compressive loading [36]. Overall, increasing the number of unit cells enhances the elastic modulus of the rhombicuboctahedron structures, while maintaining nearly constant specific compressive strength. This demonstrates that the unit cell-based architecture exhibits scalable and mechanically consistent behavior, allowing the structure to be expanded without introducing efficiency loss, and thus enabling further performance tuning through geometric modification.

### 3.2. Compressive Behavior According to Rhombicuboctahedron Types

**Figure 2:**
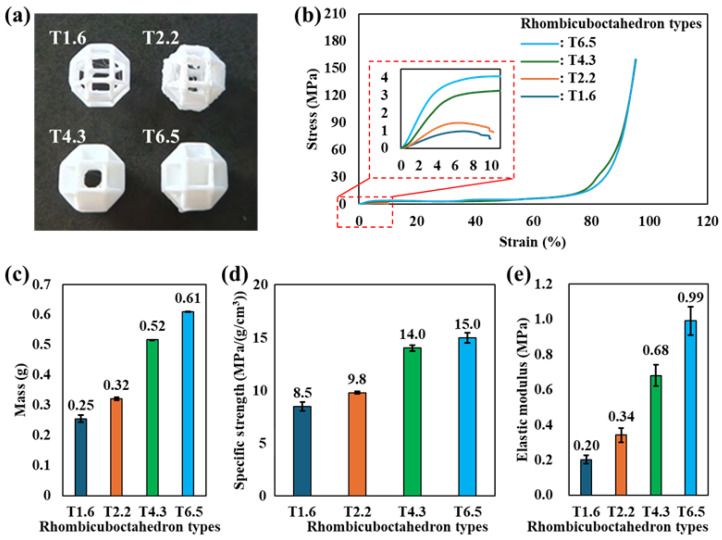
(**a**) Images of MEXed specimens, (**b**) stress–strain curve, and (**c**) comparison of mass, (**d**) specific strength, and (**e**) elastic modulus for rhombicuboctahedron structures according to the rhombicuboctahedron types.

Based on the results presented in Section 3.1, the specific compressive strength was found to be nearly independent of the number of unit cells. From a MEX process perspective, this indicates that a single-unit configuration (1 × 1 × 1) is sufficient and efficient for characterizing the unit cell mechanical response, as it minimizes material consumption and processing time while preserving stress-normalized behavior. Accordingly, a 1 × 1 × 1 rhombicuboctahedron structure was selected as the baseline geometry, and the compressive behavior was systematically investigated by varying the strut thickness (rhombicuboctahedron types) as illustrated in Figure 2a. Figure 2b shows the stress–strain responses of the rhombicuboctahedron structures with different strut thicknesses. As the strut thickness increased from T1.6 to T6.5, a pronounced enhancement in compressive strength was observed over the entire strain range. In particular, the specimens with thicker struts (types T4.3 and T6.5) did not exhibit a distinct stress drop or abrupt failure within the measured strain range. Instead, the compressive stress continued to increase with strain, indicating a transition from a deformation-dominated lattice response to a progressively densified, solid-like load-bearing behavior [37,38]. For thinner struts, deformation is primarily governed by bending and local instability of individual strut members, leading to an earlier onset of nonlinearity. In contrast, thicker struts promote load transfer through axial compression and mutual constraint at the junctions, thereby suppressing localized failure and enabling continuous stress accumulation through progressive densification rather than catastrophic fracture. Thus, the absence of stress drop in the thicker strut configurations implies that bending-induced buckling does not occur during compression. As the strut thickness increases, the cross-sectional moment of inertia of each member rises significantly, which increases the critical buckling resistance of the struts. At the same time, the reduced rotational freedom at the junctions due to the thicker connections promotes load transfer through axial compression rather than bending. Under this condition, the applied load is not concentrated in a limited number of slender members but is naturally distributed through multiple interconnected struts within the unit cell. This behavior corresponds to the formation of multiple load transfer paths and the suppression of local buckling within the rhombicuboctahedron architecture. Specifically, the rapid stress increasing shown in Figure 2d can be interpreted using the Gibson–Ashby relationship σ* ∝ ($\rho^{*}/\rho_{s}$)*^n^*, where σ* is the compressive strength of the lattice structure, $\rho^{*}$ is the density of the lattice structure, $\rho_{s}$ is the density of solid PLA, and *n* depends on the deformation mode [16,19]. Thus, the thicker strut configurations do not exhibit collapse or brittle fracture, but instead can show increasing stress with continuously progressive densification of the lattice structure due to compression. The effect of strut thickness on structural mass is summarized in Figure 2c. As expected, the mass increased monotonically with strut thickness due to the increased material volume within the unit cell. As shown in Figure 2d, the specific compressive strength was calculated using the maximum stress before the plateau region. Specifically, the specific compressive strength increased by approximately 1.76 times as the rhombicuboctahedron type changed from T1.6 to T6.5, indicating that geometric densification within a fixed unit cell volume is an effective strategy for enhancing load-bearing efficiency. The CV% of the specific compressive strength was 5.14%, 1.05%, 2.01%, and 3.09% for T1.6, T2.2, T4.3, and T6.5, respectively. A one-way ANOVA confirmed that the differences between the rhombicuboctahedron types were statistically significant (F = 245.08, *p* < 0.001). The elastic modulus extracted from the initial linear region of the stress–strain curves in Figure 2b is presented in Figure 2e. The elastic modulus increased by approximately 4.95 times as the rhombicuboctahedron type increased from T1.6 to T6.5, reflecting the enhanced stiffness associated with thicker load-bearing members. A similar interpretation can be extended to the elastic modulus shown in Figure 2e using the Gibson–Ashby relationship $E^{*}/E_{s}\propto (\rho^{*}/\rho_{s}{)}^{m}$, where $E^{*}$ and $E_{s}$ are the elastic modulus of the lattice structure and solid PLA, respectively [16,19]. This relationship suggests that, as the effective material fraction within the unit cell increases with strut thickening, the stiffness of the lattice structure correspondingly increases, which is consistent with the elastic modulus trend observed in Figure 2e. These results confirm that strut thickening effectively increases both the elastic resistance and the compressive load capacity of the rhombicuboctahedron structure. The CV% of the elastic modulus was 12.32%, 11.70%, 8.82%, and 8.07% for T1.6, T2.2, T4.3, and T6.5, respectively. A one-way ANOVA confirmed that the differences between the rhombicuboctahedron types were statistically significant (F = 122.70, *p* < 0.001). Overall, these results demonstrate that once the unit cell size is fixed, strut thickness becomes the dominant geometric parameter governing compressive strength, stiffness, and deformation mode. The absence of abrupt failure and the emergence of progressive densification behavior in thicker rhombicuboctahedron types highlight the potential of this architecture for applications requiring stable and predictable compressive performance under large deformation.

### 3.3. Feasibility Test

To demonstrate the feasibility of the rhombicuboctahedron structure for practical lightweight applications, the specific compressive strength of lattice structures fabricated by the PLA-MEX was quantitatively compared. The BCC and TLC lattice structures have been reported to be less than 1.5 MPa/(g/cm^3^) and less than 11 MPa/(g/cm^3^), respectively [39]. Furthermore, Gyroid lattice structures fabricated by varying the wall thickness and cell size have been reported to have specific strengths in the range of approximately 4.4–14.0 MPa/(g/cm^3^) [40]. In addition, co-continuous interlocking PDMS/PLA composite lattice structures have been investigated to further enhance mechanical performance during compression. The specific strength strongly depended on the PLA strut diameter, where specimens with strut diameters of 3, 5, and 7 mm exhibited specific strengths of approximately 2.9, 8.26, and 17.1 MPa/(g/cm^3^), respectively. This demonstrates a representative case in which the combination of two materials can increase the specific strength [41]. Finally, an ultra-high-specific-strength lattice, referred to as the X-lattice, has recently been developed. This newly proposed lattice structure, fabricated using PLA-MEX, exhibited a specific compressive strength of approximately 53.6 MPa/(g/cm^3^). Notably, this value was reported to exceed the specific strength levels of several metallic lattice structures [42].

**Figure 3:**
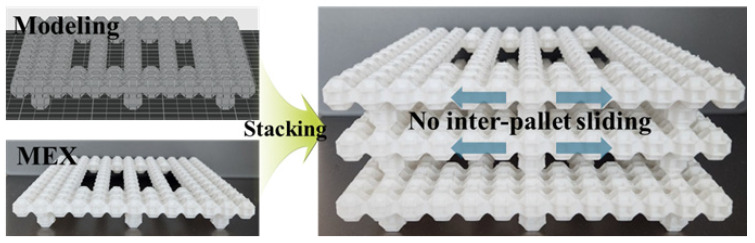
Feasibility demonstration of pallet structure using rhombicuboctahedron.

Therefore, the T6.5 rhombicuboctahedron structure reported in this study, exhibiting a relatively high specific compressive strength of approximately 15.0 MPa/(g/cm^3^), can be considered a suitable unit cell design for pallet-type structural applications subjected primarily to compressive loading during service [43]. Unlike conventional pallet structures based on simple cubic or planar geometries, the proposed architecture is not strictly confined within a cubic envelope. As a result, when multiple pallets are stacked, the unit cells partially interlock with each other through their recessed polyhedral geometry as shown in Figure 3. This geometric interlocking effect effectively suppresses lateral sliding between stacked pallets, thereby enhancing stacking stability. For a conventional flat pallet with planar contact, the onset of sliding on an inclined surface can be described by a simple frictional model. When a pallet is tilted by an angle θ, sliding begins when the downslope component of the gravitational force exceeds the maximum static friction force at the interface, i.e., $mgsin\;\theta =\mu_{s}mgcos\;\theta$. This leads to the relation $tan\;\theta_{\mathrm{slip}}=\mu_{s}$, where $\mu_{s}$ is the static friction coefficient between contacting surfaces. Using a literature-reported static friction coefficient for PLA of approximately $\mu_{s}\approx 0.30$ [44], the theoretical slip-initiation angle for a conventional flat pallet is estimated as $\theta_{\mathrm{slip}}\approx 17^{\circ }$. In contrast, the proposed rhombicuboctahedron pallet does not rely solely on frictional resistance. The 45° recessed facets allow geometric interlocking between stacked pallets. As a conceptual estimate, this interlocking interface can be approximated as a wedge-type contact with angle $\alpha \approx 45^{\circ }$, for which an effective resistance to sliding can be expressed as $\mu_{\mathrm{eff}}\approx (\mu_{s}+tan\;\alpha )/(1-\mu_{s}tan\;\alpha )$ [45]. Substituting $\mu_{s}\approx 0.30$ gives $\mu_{\mathrm{eff}}\approx 1.86$, corresponding to an estimated slip-initiation angle of $\theta_{\mathrm{slip}}\approx 62^{\circ }$ (since tan $\theta_{\mathrm{slip}}$ = $\mu_{\mathrm{eff}}$). This value should be interpreted as an idealized theoretical estimate illustrating the strong geometric contribution of interlocking, rather than as an experimentally verified performance limit.

In addition, the non-planar surface topology formed by the rhombicuboctahedron units introduces multiple surface asperities and recessed regions. When objects are placed on the pallet, these geometric features increase the frictional resistance at the contact interface by inducing localized mechanical constraints and elevated contact pressures. Consequently, the proposed pallet structure is expected to provide improved anti-slip performance compared to conventional flat-surface pallets, while maintaining lightweight and load-bearing efficiency. These characteristics demonstrate that the rhombicuboctahedron architecture is well-suited for pallet applications requiring lightweight design, stable stacking, and enhanced resistance to sliding under compressive loading.

## 4. Conclusions

In this study, the geometric and compressive characteristics of MEXed rhombicuboctahedron structures were systematically investigated. The compressive results showed that increasing the number of unit cells led to the specific compressive strength remaining nearly constant. Furthermore, thicker struts significantly increased both the elastic modulus and the specific compressive strength, while suppressing premature fracture. In particular, the thickest configuration exhibited progressive densification behavior with continuously increasing stress, indicating a stable and solid-like load-bearing response under compression. Based on these mechanical characteristics, the optimized rhombicuboctahedron structure was applied to a pallet-type configuration to demonstrate practical feasibility. Overall, this work demonstrates that rhombicuboctahedron-based architectures fabricated by MEX can provide scalable, efficient, and stable load-bearing performance. The findings offer fundamental insights into geometry-driven structural design and suggest promising opportunities for applying polyhedral lattice architectures to practical engineering components subjected to compressive loading. However, this study is limited to a single material, the MEX process, and a compressive loading condition. Future work will extend this investigation to broader materials, geometries, MEX process parameters, and loading scenarios. Furthermore, as the pallet application presented in this study is primarily conceptual, future work will include a quantitative evaluation of slip resistance by measuring friction coefficients using appropriate standardized test methods.

## Data Availability

The original contributions presented in this study are included in the article. Further inquiries can be directed to the corresponding author.
